# Managing pediatric thoracic outlet syndrome

**DOI:** 10.1186/s13052-015-0128-4

**Published:** 2015-03-27

**Authors:** Aierken Rehemutula, Li Zhang, Lin Chen, Desong Chen, Yudong Gu

**Affiliations:** Department of Hand Surgery, Huashan Hospital, Fudan University, Shanghai, 200040 People’s Republic of China; Key Laboratory of Hand Reconstruction, Ministry of Health, Shanghai, 200032 People’s Republic of China; Shanghai Key Laboratory of Peripheral Nerve and Microsurgery, Shanghai, 200032 People’s Republic of China

**Keywords:** Brachial plexus, Cervical rib, Hypesthesia, Neck pain, Radial artery, Thoracic outlet syndrome

## Abstract

**Background:**

Thoracic outlet syndrome (TOS) is largely overlooked in children and adolescents because the condition is not widely viewed as a pediatric disorder. This study aimed to clarify the causes, best treatment approaches, and prognosis for young patients with TOS.

**Methods:**

A retrospective study was conducted on 13 patients, from 4 to 13 years of age, with TOS. Ten children underwent surgical treatment, and three were treated conservatively. All patients received local nerve blocks on two occasions and were followed-up for more than 2 years.

**Results:**

Among the 10 children who underwent surgery, six school-aged children returned to school 10 to 14 days after surgery. Parents of the three children treated conservatively reported that activity within the affected limb and overall muscle strength had increased in their children and none of the three children had complained about discomfort in the affected limb.

**Conclusion:**

A diagnosis of TOS should be considered when a child or adolescent has neck and shoulder discomfort, hand numbness, and upper limb weakness. As with adults with TOS, detailed physical examination is the key to diagnosing pediatric TOS. Conservative treatment is effective for young TOS patients who have mild changes in the length and thickness of the affected limb and is an option when parents refuse surgical treatment.

## Introduction

Thoracic outlet syndrome (TOS), or brachial plexopathy, is one of the most common types of neurovascular bundle compression disorders. Most cases involve the inferior trunk of the brachial plexus, usually among middle-aged women. The male to female ratio is 1:3, and more than 80% of patients are between the ages of 25 and 40 years [1].

TOS also occurs in young children and teenagers, but the diagnosis is often missed because TOS is not commonly thought of as a disorder that affects children. In addition, children are likely to attribute their symptoms to simple muscle strain [2].

There is still controversy concerning the definition of TOS because the term only outlines the location of the problem without defining its pathogenesis [3]. To date, there have been only a few case reports, a few descriptions of anatomic abnormalities, and only a few short-term follow-up studies of TOS in children and teenagers. Thus, there is little information available to analyze the etiology, treatment options, and outcomes of TOS among younger patients. Thus young patients may be treated conservatively rather than receive surgical intervention [4].

The main clinical signs of TOS in adults include ipsilateral upper limb pain and discomfort, weakness, cold intolerance, and numbness of the hand. During physical examination, the muscles of the ipsilateral limb are relatively weak, and acmesthesia, or pinprick sensation without pain, is present on the inner surface of the hand and forearm. Thenar and hypothenar muscle atrophy may also be seen.

In contrast, in children and teenagers, TOS usually presents as neck discomfort, upper limb numbness, weakness, and sensory loss. In one study of 35 adolescents, 18 had venous symptoms, nine had neurogenic symptoms, eight had arterial symptoms, and five had abnormal ribs. In that study, adolescents more often presented with the venous and arterial forms of TOS compared with the adult presentation of the disorder [5]. Other authors have reported that the same symptoms and thrombotic complications occur in pediatric patients as in adults, and, thus, the same surgical strategy is effective in adults and children [6]. Some younger patients report that the affected limb “feels smaller” than the other arm. Often parents, teachers, and even clinicians think these symptoms are merely the result of poor posture, or these children are thought to have cervical spondylosis or neuroses.

TOS is divided into three types including neurogenic, the most common type (about 95% of cases), arterial, and venous TOS (about 5% of cases) [7-9]. Symptoms and signs of neurogenic TOS include hand numbness, weakness, and muscle atrophy. Patients with arterial TOS have low skin temperature, weakness, and dark coloration of the hand. However, patients with venous TOS have symptoms such as swelling of the hand and increasing pain.

Although the underlying cause in many TOS cases is unknown, the most widely recognized cause of TOS is an elongated transverse process of C7 which compresses the brachial plexus. TOS is generally believed to result from one of two major factors, compression and infection. There is also a paucity of information concerning the course of TOS among younger patients. This study was designed, therefore, to explore the causes of the disease and best treatments, as well as the prognosis in young patients with TOS. In most studies, the main treatment option has been surgery, but in this retrospective study, both surgical and nonsurgical treatments were explored, and all patients were followed-up from 6 months to 2 years.

## Materials and methods

### Patients

Our study protocol was approved by Huashan Institutional Review Board (HIRB). Written informed consent was obtained from the parents of every child prior to their participation in the study.

All children under the age of 18 with TOS who underwent treatment for TOS at our hospital between January 2008 and June 2014 were enrolled in the study.

The inclusion criteria were: (1) typical clinical presentations of TOS; (2) positive results on Wright’s and Adson’s tests, and a positive Hoffmann’s reflex; (3) electromyographic (EMG) findings supporting a diagnosis of TOS; and (4) age younger than 18 years. Children with congenital brachial plexus injury, obstetric brachial plexus palsy, congenital torticollis, endocrine diseases (such as peripheral neuropathy caused by pediatric diabetes), or a recent history of trauma were excluded from the study.

A final group of 13 patients (3 boys and 10 girls) from 4 to 13 years of age (mean age, 8 years) who met our criteria were included in the study.

### Physical examination

We studied test results, radiographs, and EMG results from all patients, and all patients underwent Wright’s test, Adson’s test, and a test to elicit Hoffmann’s sign.

#### Hyperabduction maneuver (Wright’s test)

With the patient seated, the radial pulse is palpated. Then the patient’s shoulder is placed into abduction and externally rotated 90 to 100 degrees while the elbow is flexed to 90 degrees. A change in the radial pulse (disappearance or weakening of the radial pulse) is considered to be a positive result. This examination has a very high positive rate; however, it also has a relatively high false-positive rate.

#### Adson’s test

As in the Wright’s test, the radial pulse is palpated while the patient is seated. Then, the patient’s shoulder is abducted to 30 degrees, with slight extension. The patient then hyperextends his or her neck and turns the head toward the ipsilateral side. Disappearance of, or weakening of, the radial pulse is considered to be a positive result. Although only 14% of patients with TOS have positive responses, Adson’s test is often diagnostic.

#### Hoffman’s sign

Applying light percussion or by flicking specific nerves in the fingers or the wrists causes a tingling sensation that may point to nervous system irritation.

### Treatment

If an elongated transverse process was observed on the plain radiographs, surgical treatment was indicated. If no bony lesion was found on the radiographs, but there were changes on the EMG examination, a local block was used and a local anesthetic and a steroid were administered.

#### Conservative treatment

Patients with early-stage TOS were treated with rest and instructions concerning proper posture, i.e., patients were advised to avoid heavy physical labor and strenuous activity, and were instructed to cross their arms on their chest while slightly raising both shoulders, to relax the brachial plexus. For patients with serious discomfort in the neck, a local nerve block was applied at the site of tenderness. This local nerve block relieved the localized pain caused by injecting local anesthetics. In addition, the steroids prolonged the nerve block and eliminated inflammation, leading to excellent outcomes in pain relief and healing.

Once a week, patients received an injection of 2 mL triamcinolone acetate plus 2 mL 0.5% bupivacaine and this treatment was repeated for 4 to 6 consecutive weeks. Neurotropic drugs, such as vitamin B1 (thiamine), vitamin B2 (riboflavin), or bendazole, were simultaneously administrated. Cervical traction was likely effective in some patients because the traction released the cervical muscles and thus reduced compression on the brachial plexus.

#### Surgical treatment

Surgery was offered to patients with the following symptoms: (1) limb and neck discomfort that was affecting their quality of life; (2) decreased muscle strength and muscle atrophy or upper limb motor dysfunction; and (3) significantly decreased sensation within the hand and acmesthesia.

Several surgical procedures were available to the children. Scalenectomy was suitable for all TOS patients who did not have bony compression.

Once the scalenus anterior, scalenus medius, and scalenus minimus muscles are removed, compression from the superior, inferior and bilateral sides lessens or even disappears. Therefore, this procedure, the most common surgical treatment for TOS, can be used in patients with various types of the disease. The procedure can be performed through a 7- to 8-cm transverse incision made in the lower neck. Some portion of the hypertrophic scalene muscle can be removed. For patients with neck and shoulder pain or C5 compression, the origins of the scalenus anterior and scalenus medius near C5 and C6 should be excised.

When radiographs of the cervical spine revealed a cervical rib, the rib was removed after excising the scalenus anterior, scalenus medius, and scalenus minimus.

A third surgical option involves resection of the transverse process of C7. If the transverse process of the seventh cervical vertebra is longer than that of the first thoracic vertebra, the former should be removed.

Another surgical approach involves resection of the first rib. Before removing the first rib via an incision through the neck, the insertions of the scalenus anterior and scalenus medius muscles should be excised, followed by subperiosteal resection of the first rib. Good candidates for this procedure include patients without significant bone compression, but with considerable scalene abnormalities and an abnormal band compressing the brachial plexus.

Some of the surgical complications that can occur among adult and pediatric patients include brachial plexus injury, pneumothorax, chylous leakage, lymph effusion, and hematomas. One week of temporary immobilization was applied to our 4-6 year old pediatric patients, using either external fixator or cast, to prevent lymphorrhagia and hemorrhage caused by noncompliance with treatment.

## Results

The clinical characteristics of the 13 children (three males and 10 females; mean age, 8 years of age) included in the final study group are shown in Table 1. The youngest child was 4 years old and the eldest was 13 years old. The child’s parents had detected the disease as the disease had gradually worsened in each child. The longest course of disease was 4 years, and the shortest was 2 years.

**Table 1:** **Characteristics of 13 children with TOS**

**Patient**	**Sex**	**Age (yr)**	**Side**	**Type**	**Tests**	**Anatomic anomalies**	**Treatment**	**Follow-up**	**Complication**
1	F	4	Left	Neurogenic	Wright test (+), Adson test (+)	C5-T1 nerve roots and the upper, middle, and lower trunks of the brachial plexus were wrapped by proliferated connective tissue.	Surgery + Local block	1Y	Pain, improved after Localblock
2	F	6	Both	Neurogenic	Negative	Lymphatic vessels and lymph nodes were found in the fat pad of the posterior cervical triangle.	Surgery	18 M	No
3	M	6	Left	Neurogenic	Wright test (+),Adson test (+)	The front edge of scalenus anterior, scalenus medius, and scalenus minimus were composed entirely of tendon tissue.	Surgery	3Y	No
4	M	6	Right	Neurogenic	Negative	Nonsurgical treatment	Local block	2Y	No
5	F	6	Right	Neurogenic	Negative	Nonsurgical treatment	Local block	2Y	No
6	F	7	Both	Neurogenic	Wright test (+), Adson test (+)	Nonsurgical treatment	Local block	2Y	No
7	F	7	Left	Neurogenic	Negative	Lymphatic vessels and lymph nodes were found in the fat pad of the posterior cervical triangle.	Surgery	18 M	No
8	F	7	Right	Arterial	Wright test (+),Adson test (+)	Lymphatic vessels and lymph nodes were found in the fat pad of the posterior cervical triangle.	Surgery	2Y	No
9	M	9	Right	Neurogenic	Wright test (+),Adson test (+)	Lymphatic vessels and lymph nodes were found in the fat pad of the posterior cervical triangle.	Surgery + Local block	2Y	No
10	F	9	Left	Neurogenic	Wright test (+),Adson test (+)	The front edge of scalenus anterior, scalenus medius, and scalenus minimus were composed entirely of tendon tissue.	Surgery + Local block	20 M	No
11	F	11	Left	Neurogenic	Wright test (+),Adson test (+)	C8 and T1 nerve roots were wrapped by proliferated connective tissue.	Surgery	14 M	No
12	F	13	Right	Arterial	Wright test (+),Adson test (+)	The front edge of the scalenus anterior, scalenus medius, and scalenus minimus were composed entirely of tendon tissue.	Surgery + Local block	16 M	No
13	F	13	Left	Venous	Negative	The front edge of the scalenus anterior, scalenus medius, and scalenus minimus were composed entirely of tendon tissue.	Surgery + Local block	18 M	No

TOS involved the left side in six patients, the right side in five patients, and both sides in two patients. All 13 children had neck pain or discomfort, and ipsilateral limb pain and weakness. The youngest patient, a 4-year-old girl, complained of left upper limb pain, which she had experienced since 2 years of age. The girl often cried at night and could sleep only 3 to 4 hours a night. One 9-year-old boy complained of neck discomfort and difficulty writing; he had noticeable right upper limb discomfort after writing longer than 30 minutes. A 13-year-old girl had a two-year history of right upper limb pain and weakness. Her condition had been diagnosed as a “neurosis” by a physician, and she was once considered to be malingering when she did not go to school. Other children were admitted to our hospital with neck discomfort and limb weakness. Eleven of the 13 children in our study had been diagnosed as having cervical disease; two children had been previously diagnosed as having neuroses, and one patient had been diagnosed as having a congenital disorder.

**Figure 1:**
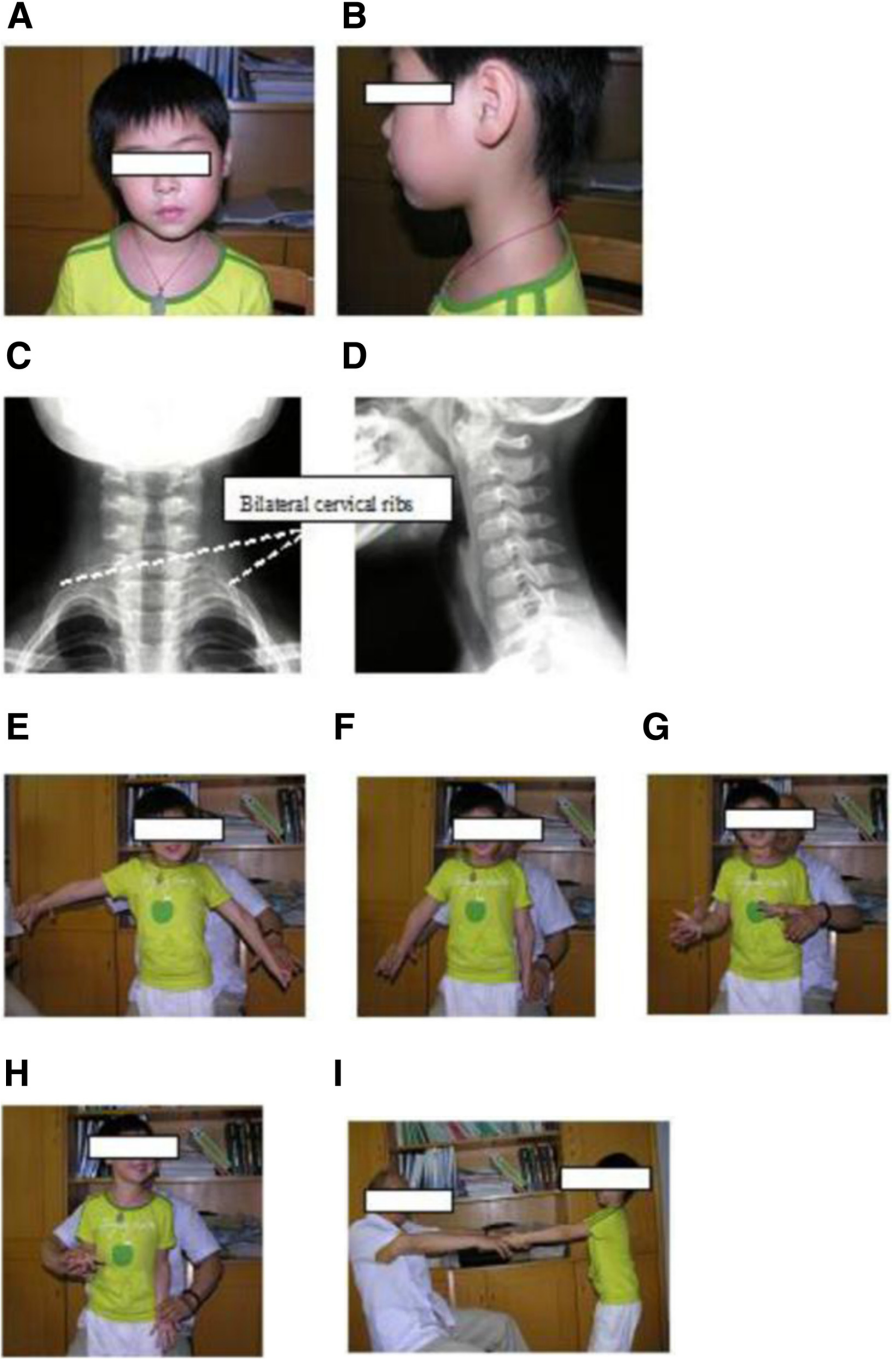
**A 6-year-old girl had neck, shoulder and bilateral upper limb pain and discomfort for more than 2 years.** She had complained of left upper limb pain since she was 3 years old. **(A)** On examination, the left side of the girl’s face is obviously larger than the right side and **(B)** she reported that her neck was stiff. Bilateral cervical ribs can be seen on an anteroposterior X-ray film of the cervical spine **(C)**, and a lateral X-ray film of the cervical spine shows disappearance of the physiological curvature of the cervical spine **(D)**. After receiving a local nerve block in the neck, the girl was alert, agile, and cooperative during physical examination. The shoulder abduction and external rotation strength, elbow flexion strength and hand-clenching strength of the left upper limb were poorer than those of the right upper limb. Wright’s test and Adson’s test were positive, and Hoffmann’s sign was negative. Her left shoulder abduction strength decreased at 90 degrees **(E)**. Her left shoulder abduction strength was decreased at 30 degrees **(F)**, and the left shoulder external rotation strength was also decreased **(G)**. Her elbow flexion strength was decreased **(H)**, as was her hand grip strength **(I)**. After she received a local nerve block, her shoulder, elbow and hand strength increased significantly and the acmesthesia noticeably improved.

After receiving a local nerve block in the neck, all 13 children were alert, agile, and cooperative during their physical examination. During the physical examination, we noticed that the affected limbs were relatively thin and short compared with the contralateral limbs. In three cases, the length of the affected limb (measured from the elbow to the tip of the middle finger) was 2 cm to 3 cm shorter than that of the healthy limb. Another feature observed in these three children was facial asymmetry (Figure 1A).

In nine children, acmesthesia was decreased along the medial side of the entire upper limb. In four cases, acmesthesia was decreased along the medial side of the hand and forearm. In all cases, shoulder abduction and external rotation strength, elbow flexion strength and hand-clenching strength of the affected limb were poorer compared with those of the contralateral limb.

Cervical spine radiographs showed that the physiological curvature of the cervical spine disappeared in one 6-year-old boy, and movement of his neck was subsequently affected. The child found it impossible to extend his neck and could not lower his chin to his chest. In another case, the transverse process of C7 was relatively large. One child had bilateral cervical ribs. No other abnormalities were observed among the other children.

On detailed physical examination, eight children had positive results on Wright’s test, and one child had a positive Adson’s test. In all cases, the radial artery pulse disappeared when the subclavian artery was compressed. In one child, when the subclavian artery pulse was gently touched 3 cm superior to the clavicle at the posterior margin of the sternocleidomastoid, the radial artery pulse disappeared.

### Conservative treatment

Two females and one male child underwent conservative treatment. One approach addressed posture during writing because it was noted that four children skewed their heads to the right while they were writing. Correcting their writing posture became very important because these children regularly spent 3 to 4 hours writing each day which greatly affected the tension in the cervical muscles and the children’s vision. After we instructed the parents, they supervised their children while the children did 200 to 400 shoulder shrugs over several sessions (with 5 minutes of rest between each session). This exercise increased the power of the trapezius muscle and reduced the traction of the sagging upper limb on the brachial plexus. In addition, a local nerve block with 0.5 mL betamethasone dipropionate (Diprospan®, Schering-Plough Labo, Brussels, Belgium) or 10 to 20 mg/mL triamcinolone acetonide + 2 mL of 0.25% bupivacaine (or 2 mL of 0.25% ropivacaine) were used as local nerve blocks. Additional local nerve blocks were performed for patients with decreased sensory and muscle power.

### Surgical procedures and intraoperative findings

Ten children underwent surgery. After undergoing general anesthesia, the patient was placed in the supine position, with a pillow beneath the shoulder and with the head turned to the opposite side. During surgery, many large lymph nodes were found in the fat pad of the posterior cervical triangle. The largest lymph node measured 2 cm × 1 cm × 1 cm. Lymph node biopsies were performed in four patients, and all showed chronic inflammation. The scalenus anterior and scalenus medius muscles were relatively tense and hypertrophic and were excised at the level of the subclavian artery. The muscle fibers of the scalenus anterior and scalenus medius muscles, near the C5 and C6 nerve roots, were also excised.

During surgery, it was observed that all the children had varying degrees of proliferation within the outer membrane of the brachial artery. Three patients had relatively white tenuous connective tissue on the surface of the scalenus anterior and scalenus medius muscles. The results of pathological examination revealed hyperplasia of collagen and fibrous tissues. During surgery on a 4-year-old girl who had mainly complained of pain, it was found that the C5 to T1 nerve roots and the upper, middle, and lower trunks of the brachial plexus were wrapped in proliferative, and relatively hard, connective tissue. After removing the surrounding connective tissue, the residual texture of the nerve trunk was relatively good. In seven patients, there was an obvious arc at the site of the inferior trunk of the brachial plexus, or at the site where C8 to T1 nerve roots cross the scalene minimus. The scalene minimus at this location was composed of tendon or fascial tissue. In a 9-year-old boy, the anterior margin of the scalene minimus was completely composed of tendon tissue. The subclavian artery was elevated significantly in four patients.

Branches originating from the subclavian artery to the inner and outer sides of the brachial plexus were cut off, together with branches passing through the brachial nerve, and then the scalenus anterior, scalenus medius and scalenus minimus muscles were excised. A solution of 2.5 mL to 3 mL of triamcinolone acetonide was diluted to 5 mL to 6 mL using 2% lidocaine, and then injected into the outer membrane of the exposed brachial plexus.

Cervical nerve blocks and diagnostic treatment were performed in 11 children. After a local block, the muscle strength of the shoulder, elbow and hand increased significantly, and the acmesthesia improved markedly.

A 6-year-old girl underwent surgery due to discomfort and weakness in the right upper limb. The discomfort of the right upper limb disappeared after surgery, and her muscle strength increased. However, discomfort and weakness in the left upper limb remained, together with dysesthesia, and the parents approved surgery for the left upper limb. Preoperative examination revealed no significant differences in thickness and length between both upper limbs. However, 18 months prior to this, the right upper limb had been relatively thin and short compared with the left upper limb. The discomfort and weakness of the left upper limb disappeared after surgery.

After surgery, all children received 5-mg intravenous injections of dexamethasone. Injections of 10 mg vitamin B6 and 10 mg vitamin B1 were administered three times a day for two weeks. There were no complications such as pneumothorax, wound hematoma, chylous leakage, or lymph effusion.

After surgery, we instructed parents to help their children perform exercises such as raising the affected limb, flexing the elbow, and grasping toys with the hand on the affected side. For children with relatively significant differences in the thickness and length of the bilateral upper limbs, we bound the healthy limb to the trunk, and then had the children play games using only the affected upper limb.

### Follow-up results

Follow-up duration ranged from 6 to 30 months. The disease was controlled satisfactorily in the three children who received conservative treatment. All patients had local nerve blocks on two occasions and were observed for more than 2 years. Among the 10 children who underwent surgery, six children were able to return to school 10 to 14 days after surgery. Parents of three children reported that the muscle strength of the affected limb had increased, and none of the three children complained of discomfort in the affected limb. One 9-year-old child and two 7-year old children told their parents that the strength of their affected limb had increased and became similar to the healthy limb after surgery. Only the 4-year-old girl, who had mainly complained of left upper limb pain, had cried at night 5 to 6 days after surgery. Two months later we administered an infraclavicular brachial plexus block, and within 2 weeks, her pain had gradually disappeared.

## Discussion

Conservative treatment is effective for children with TOS who have mild changes in the length and thickness of the affected limb. It is also an option when parents are adverse to surgical treatment for their child. Conservative treatment is aimed primarily at correcting posture during reading, writing, and watching television. This relieves compression from the peripheral tissue on the brachial plexus. Appropriate cervical traction can further relax the cervical muscles, and cervical nerve block can soften the scalenus anterior and scalenus medius muscles. These treatment methods are effective in stabilizing pediatric TOS. The key point of conservative treatment is persistence. The three children who received conservative treatment all had satisfactory outcomes. We are continuing our follow-up of these patients.

Surgical treatment of pediatric TOS is considered an absolute solution aimed at the cause of the disease. Of course, postoperative active rehabilitation and strength training of the affected limb are also essential. Because children grow continuously, development of the affected limb can be affected as soon as the brachial plexus is compressed. Therefore, early decompression is very important.

Inflammatory cytokines stimulate the scalenus anterior, scalenus medius, and scalenus minimus muscles to induce spasm within these muscles and promote proliferation of peripheral connective tissue which compresses the brachial plexus. If the proportion of tendon near the nerve is high in the scalenus muscles, the brachial plexus is more easily compressed. Thus, early relief of the peripheral tissue compressing the brachial plexus and active postoperative rehabilitation are very important.

As Howard et al. have reported, diagnosing and documenting brachial plexus compression in TOS is difficult because the syndrome overlaps with cervical disc disease, intrinsic shoulder pathology, and peripheral nerve compression, and traditional electrodiagnostic testing cannot identify brachial plexus compression in the thoracic outlet [10].

Seven patients in our study had varying degrees of thinning of the affected limb. Among them, the muscle strength of the affected limb decreased significantly in one 9-year-old boy. We administered a single cervical nerve block to him in the outpatient department and the immediate outcome was satisfactory. His muscle strength increased and his sensory ability improved. However, the improvement only lasted about 12 hours. The satisfactory results from the local nerve block gave us confidence to pursue a surgical approach. After obtaining parental consent, we excised the scalenus anterior and scalenus medius together with partial excision of fibers of the scalenus anterior, scalenus medius, and scalenus minimus near the C5 and C6 nerve roots. After surgery, the boy started strength training of the affected limb. When he visited our hospital 6 months later, the upper limb thickness, muscle strength, and degree of acmesthesia were similar in both limbs and the boy reported that he could now write without feeling tired.

In studies of surgical treatment among 11 young TOS patients, three children had cervical ribs removed [2,6]. After resection of the cervical rib, the upper limb discomfort disappeared and their muscle strength increased. In one patient, a subclavian aneurysm shrank after removal of the pseudoarthrosis which had formed near the first and second ribs. The scalene muscles were removed from the first rib in other cases. Symptom relief was observed in six out of seven children although one patient had a recurrence of symptoms. In yet another study of 322 children, 88.8% were asymptomatic and the most common symptoms were neck mass and pain. In that series, the most useful diagnostic tools were the cervical spine and chest radiographs [11].

Our study had several limitations including its retrospective nature and small sample size. In addition, the follow-up data were incomplete and there was no long-term follow-up. We also were unable to establish a standard procedure for conservative treatment, but tailored treatment to the individual child. Because of their young age, compliance with exercise and self-care was not generally high among the children.

Much still remains to be learned concerning TOS in pediatric patients. In our small study, we found that some children benefited from conservative treatment, and we were able to relieve symptoms in all 13 patients. We are continuing to follow-up this group of patients.

## Conclusion

Pediatric TOS may be related to acute or chronic infection of the cervical lymph nodes and subsequent cervical inflammation. A diagnosis of TOS should be considered when a child or adolescent has neck and shoulder discomfort, hand numbness, and upper limb weakness. As with adults with TOS, detailed physical examination is the key to diagnosis of the pediatric form of the disease. After a definitive diagnosis is made, and when conservative treatment fails, surgery should be performed. Any delay in treatment should be avoided. It is particularly important for parents and clinicians not to arbitrarily conclude that this very real condition is malingering when a child (who does not like studying) does poorly in school.

## Abbreviations

mL: Milliliter
TOS: Thoracic outlet syndrome

## References

[1] Roos DB, Machleder HI (1989). Overview of thoracic outlet syndromes. Vascular disorders of the upper extremity.

[2] Maru S, Dosluoglu H, Dryjski M, Cherr G, Curl GR, Harris LM (2009). Thoracic outlet syndrome in children and young adults. Eur J Vasc Surg.

[3] Hooper TL, Denton J, McGalliard MK, Brismée J-M, Sizer PS (2010). Thoracic outlet syndrome: a controversial clinical condition. Part 1: anatomy, and clinical examination/diagnosis. J Man Manip Ther.

[4] Stansby G, Lambert D (2009). Thoracic outlet syndrome in children and young adults. Eur J Vasc Surg.

[5] Chang G, Graf E, Demos J, Roethle T, Freischlag JA (2011). Spectrum of thoracic outlet syndrome presentation in adolescents. Arch Surg.

[6] Vercellio G, Baraldini V, Gatti C, Coletti M, Cipoat L (2003). Thoracic outlet syndrome in paediatrics: clinical presentation, surgical treament, and outcome in a series of eight children. J Pediatr Surg.

[7] Hempel GK, Shutz WP, Anderson JF, Bukhari HI (1996). 770 consecutive supraclavicular first rib resections for thoracic outlet syndrome. Ann Vasc Surg.

[8] Sanders RJ (1996). Results of the surgical treatment for thoracic outlet syndrome. Semin Thorac and Cardiovasc Surg.

[9] Sanders RJ, Haug C (1991). Review of arterial thoracic outlet syndrome with report of five new instances. Surg Gynecol Obstet.

[10] Howard M, Lee C, Dellon AL (2003). Documentation of brachial plexus compression (in the thoracic inlet) utilizing provocative neurosensory and muscular testing. J Reconstr Microsurg.

[11] Chan KH, Gitomer SA, Perkins JN, Liang C, Strain JD (2013). Clinical presentation of cervical ribs in the pediatric population. J Pediatr.

